# Mapping Nordic Walking Research in Ageing-Related Populations: A Bibliometric and Topic Modeling Analysis (2006–2025)

**DOI:** 10.3390/healthcare14142061

**Published:** 2026-07-09

**Authors:** Hao Chen, Man Jiang, Jakub Kortas

**Affiliations:** 1 Gdansk University of Physical Education and Sport, 80-336 Gdansk, Poland; hao.chen@awf.gda.pl; 2 Basic Education Department, Xizang Vocational Technical College, Lhasa 850000, China; 13889010160@163.com

**Keywords:** Nordic walking, ageing-related populations, older adults, functional rehabilitation, topic modeling

## Abstract

Population ageing has increased the need for safe, accessible, and sustainable exercise strategies that support functional capacity, independence, and healthy ageing. Nordic walking has been examined across physiological, functional, clinical, and health-related contexts; however, previous research syntheses have generally focused on specific intervention effects, populations, or outcome categories, leaving the relationships among these research strands insufficiently understood. This study combined CiteSpace-based bibliometric analysis with Latent Dirichlet Allocation (LDA) topic modeling to examine the knowledge structure and thematic development of Nordic walking research in ageing-related populations from 2006 to 2025. A total of 263 publications indexed in the Web of Science Core Collection were included in the final analysis. The results showed that research activity was geographically concentrated, whereas institutional connections were relatively dispersed. Co-citation patterns linked the field with exercise science, rehabilitation, gerontology, gait analysis, and clinical health research. Keyword and temporal analyses indicated that research attention expanded from the physiological and biomechanical characterization of Nordic walking toward functional rehabilitation, chronic disease-related applications, and broader health outcomes. The seven LDA-derived topics reflected health-outcome evaluation, metabolic and cardiovascular indicators, functional rehabilitation, exercise physiology, cognitive health, body composition and muscle function, and gait-related rehabilitation. Rather than representing isolated research domains, these topics reveal an interconnected knowledge structure linking exercise-related mechanisms, functional and clinical outcomes, and broader ageing-related health concerns. Future research should improve intervention reporting, adopt population-specific outcome selection, and strengthen the linkage between exercise mechanisms and functional or health-related outcomes to support more comparable and cumulative evidence.

## 1. Introduction

Population ageing has become an important public health and rehabilitation challenge worldwide. With increasing age, older adults commonly experience declines in muscle strength, balance control, gait stability, aerobic capacity, and functional mobility. These changes can reduce independence in daily living, increase fall risk, and contribute to frailty, disability, and reduced quality of life [1,2,3]. Therefore, safe, accessible, and sustainable exercise strategies are needed to preserve mobility, support functional capacity, and promote healthy ageing [4,5,6].

Nordic walking is a walking-based exercise modality characterized by the active use of poles during locomotion. Compared with conventional walking, it requires coordinated movement of the upper limbs, trunk, and lower limbs, thereby transforming ordinary gait into a more integrated whole-body activity. The poles may provide additional mechanical support and improve perceived stability, while the active pushing action increases upper-body and trunk involvement [7,8]. These characteristics make Nordic walking a potentially suitable exercise strategy for older adults and for populations requiring low-cost, adaptable, and community-based physical activity [9,10].

Existing studies have examined Nordic walking in relation to a range of ageing-related health and rehabilitation outcomes. These include functional mobility, balance, gait performance, walking capacity, cardiorespiratory fitness, body composition, quality of life, and chronic disease-related rehabilitation. Nordic walking has also been investigated among healthy older adults and individuals with conditions such as Parkinson’s disease, cardiovascular disease, metabolic disorders, musculoskeletal limitations, and frailty-related functional decline [11,12,13]. However, this evidence remains fragmented across participant groups, intervention protocols, clinical contexts, and outcome domains. Studies differ considerably in intervention duration, weekly frequency, supervision, comparator conditions, and assessment methods. This heterogeneity complicates the synthesis of specific outcomes and makes it difficult to determine how the different research strands are conceptually related [14,15,16].

This fragmentation represents not only a methodological limitation but also a conceptual gap. Existing studies have examined physiological responses, biomechanical characteristics, functional mobility, clinical rehabilitation, cognitive health, and quality of life, but these domains have often been treated as separate research outcomes. Consequently, it remains unclear how exercise-related physiological and biomechanical adaptations are connected with functional independence, rehabilitation outcomes, cognitive health, and broader ageing-related health goals. Clarifying these relationships is important because the relevance of Nordic walking to ageing-related populations cannot be understood solely through isolated physiological or clinical outcomes. Instead, it requires an integrated consideration of exercise mechanisms, functional performance, clinical relevance, and health-related consequences [17,18].

Previous reviews and primary studies have predominantly addressed specific intervention effects, clinical populations, or selected outcome domains. Comparative studies have reported differences between Nordic walking and ordinary walking in physiological demand and gait-related parameters. Studies involving older adults and clinical populations have also reported outcome-specific findings related to functional capacity, balance and mobility, cardiometabolic indicators, body composition, quality of life, and rehabilitation. These studies provide useful evidence for particular populations and outcomes. However, the evidence has accumulated across diverse participant groups, intervention protocols, comparator conditions, and outcome measures. Consequently, it remains difficult to see how these lines of research relate to one another and how the field has developed over time. To complement this outcome-focused evidence, a combined bibliometric and topic-modeling approach is appropriate for addressing these structural and conceptual questions.

A combined bibliometric and topic-modeling approach is appropriate for addressing these structural and conceptual questions. CiteSpace can identify collaboration networks, co-citation patterns, keyword clusters, timeline structures, and burst terms, thereby revealing the explicit knowledge structure and research fronts of the field [19,20]. Latent Dirichlet Allocation (LDA) topic modeling can further identify latent semantic patterns from titles and abstracts, providing a complementary text-based account of how research themes are organized [21]. Integrating these approaches makes it possible to examine both the visible bibliographic structure and the underlying thematic organization of the literature. In the present study, these methods are used not to test a predefined theoretical model, but to interpret how Nordic walking research has expanded across physiological mechanisms, functional rehabilitation, clinical applications, cognitive health, and broader ageing-related health outcomes.

Accordingly, the present study uses bibliometric analysis and LDA-based topic modeling to examine Nordic walking research in ageing-related populations from 2006 to 2025. The study aims to: (1) describe the structural development of the field, including publication trends, major contributors, co-occurrence patterns, and intellectual foundations; (2) identify the main research hotspots and emerging fronts within the retrieved ageing-related Nordic walking corpus; (3) detect latent thematic patterns and topic relationships using LDA topic modeling; and (4) clarify how research priorities have evolved over time. Beyond providing a descriptive research map, this study seeks to offer an interpretive account of how Nordic walking research has developed from the characterization of an exercise modality toward a multidisciplinary research context involving functional capacity, rehabilitation, cognitive health, chronic disease management, and healthy ageing.

## 2. Materials and Methods

### 2.1. Search Strategy and Data Retrieval

The literature search was conducted in the Web of Science Core Collection (WoSCC) using the advanced search interface. WoSCC was selected as the sole data source to maintain consistency across the bibliometric and topic-modeling analyses. The study required a unified corpus containing standardized bibliographic records, citation links, author information, institutional affiliations, keywords, abstracts, and cited references so that publication trends, collaboration networks, keyword patterns, co-citation structures, and LDA-derived topics could be analyzed using the same set of documents. The use of a single database also reduced potential inconsistencies arising from duplicate records, differences in cited-reference formats, variations in author and institutional names, and unequal indexing practices across bibliographic databases.

**Figure 1:**
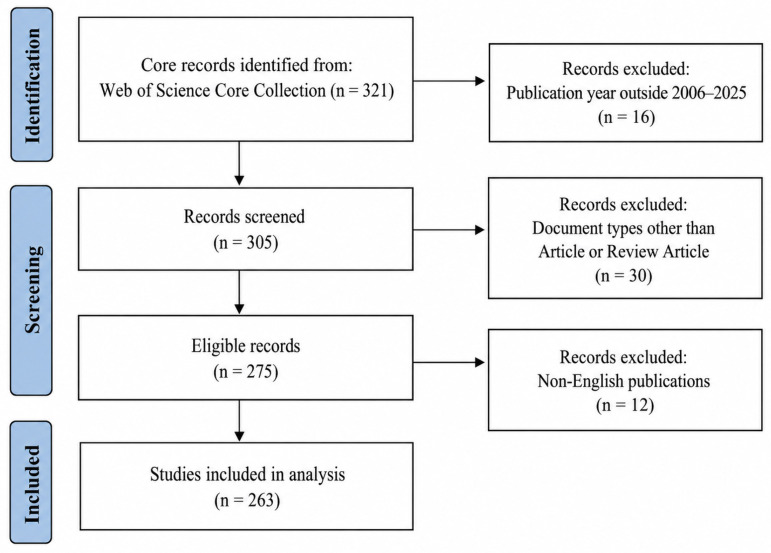
Flowchart of literature screening and study selection.

Because the purpose of this study was to map Nordic walking research in ageing-related populations, the search strategy was constructed around two concept clusters: (1) Nordic walking-related terms and (2) older adult- or ageing-related terms. Health promotion, functional rehabilitation, chronic disease management, and related clinical applications were not used as mandatory search filters. Instead, they were treated as thematic dimensions to be identified through keyword analysis, co-citation analysis, and LDA topic modeling after retrieval. This decision was made to avoid further narrowing the corpus and excluding relevant ageing-related studies that used heterogeneous terminology for outcomes such as functional mobility, balance, physical fitness, quality of life, cardiometabolic indicators, gait parameters, or rehabilitation outcomes. The search was executed on 20 March 2026 in the Topic field. The following Boolean search string was used: (“Nordic walking” OR “nordic walk*” OR “pole walking” OR “pole walk*” OR “exerstrid*”) AND (“older adult*” OR elderly OR senior* OR aged OR ageing OR aging OR “older people” OR “older person*” OR “older population*”). The initial search retrieved records related to Nordic walking and ageing populations. The initial search identified 321 records. During screening, 16 records published outside the predefined publication period of January 2006 to December 2025 were excluded, leaving 305 records. A further 30 records with document types other than Article or Review Article were excluded, resulting in 275 eligible records. After excluding 12 non-English publications, 263 publications were retained for the final CiteSpace-based bibliometric analysis and LDA topic modeling. The retrieval and screening process is summarized in Figure 1.

Eligible records were exported from WoSCC in plain-text format with full records and cited references, including bibliographic information, abstracts, keywords, affiliations, source journals, publication years, and cited references. These records formed the final dataset for CiteSpace-based bibliometric analysis and LDA topic modeling. To improve screening reliability, two authors independently assessed the retrieved records according to the predefined eligibility criteria. The initial screening agreement was 91.3%, and any discrepancies were resolved through discussion.

### 2.2. Eligibility Criteria

Records were screened according to predefined eligibility criteria. One reviewer screened titles and abstracts to exclude clearly irrelevant publications, and a second reviewer checked uncertain cases and the final included records. Publications were eligible if they focused on Nordic walking, pole walking, or exerstriding in older adults, elderly participants, or ageing-related populations. Records were retained only when sufficient bibliographic metadata, abstracts, keywords, or cited-reference information were available for bibliometric and topic-modeling analyses. Health promotion, functional mobility, rehabilitation, chronic disease management, physical fitness, and related outcomes were not applied as mandatory eligibility filters, but were examined as thematic dimensions within the retrieved corpus. Records were excluded if they were unrelated to Nordic walking, did not involve older adults or ageing-related populations, or lacked sufficient information for bibliometric or topic-modeling analysis. Any uncertainties or disagreements were resolved through discussion.

### 2.3. Data Analyses

The analysis combined CiteSpace-based bibliometric visualization with LDA topic modeling. Bibliometric analysis was used to examine publication trends, collaboration networks, co-citation patterns, keyword relationships, and research fronts, while LDA topic modeling was used to identify latent themes and their temporal changes.

The 263 eligible records were imported into CiteSpace software (version 6.3.R1; Drexel University, Philadelphia, PA, USA) for bibliometric visualization and network analysis. Analyses included country/region and institutional collaboration, keyword co-occurrence, co-citation networks, burst detection, betweenness centrality, log-likelihood ratio (LLR)-based cluster labeling, and timeline visualization. The time span was set from 2006 to 2025, with one year per time slice. The g-index selection criterion (k = 25) was applied, and the networks were pruned using the minimum spanning tree (MST) algorithm. In the resulting networks, node size represented frequency, and betweenness centrality was used to identify bridging nodes.

LDA topic modeling was implemented using Gensim and estimated using online variational Bayes. Titles and abstracts from the 263 included publications were combined to construct the document-level corpus. Text preprocessing included lowercasing, tokenization, removal of punctuation and numbers, stop-word removal, and lemmatization. General terms with limited thematic meaning were also removed where appropriate to improve topic interpretability. The processed corpus was represented as a bag-of-words document–term matrix. Candidate models with K values ranging from 2 to 10 were trained using the same parameter configuration: passes = 50, iterations = 500, alpha = 1, eta = 0.1, chunksize = 400, update_every = 1, and random_state = 42.

These candidate models were evaluated using perplexity and coherence (C_v) [22]. The final number of topics was selected by considering these indices together with the interpretability of the resulting topic structures. Topic labels were assigned by examining high-probability terms, topic-specific relevant terms, and semantic consistency. Topic strength was calculated as the mean posterior probability of each topic across documents, and temporal evolution was assessed by aggregating topic probabilities by publication year. Finally, CiteSpace results and LDA-derived topics were interpreted together to identify convergent hotspots, latent thematic structures, and changes in research priorities from 2006 to 2025.

## 3. Results

### 3.1. Publication Growth and Collaboration Landscape

**Figure 2:**
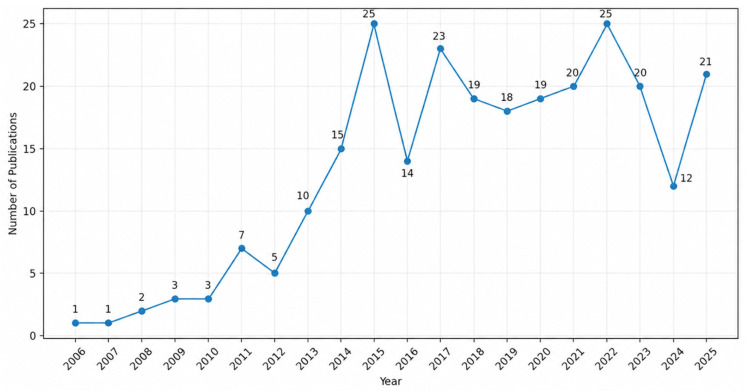
Trends in the annual number of publications from 2006 to 2025.

Figure 2 shows the annual publication trend from 2006 to 2025. The overall pattern indicates a gradual increase in research attention, but the growth was not linear. Before 2011, publication activity was limited, suggesting that Nordic walking had not yet become a stable research topic in ageing and rehabilitation contexts. A clearer expansion occurred after 2011, especially between 2013 and 2015, when annual output increased markedly. Although publication numbers fluctuated after 2015, they generally remained higher than in the early period, indicating sustained interest in Nordic walking as a health-related and rehabilitation-oriented exercise strategy. The recent rebound in 2025 after a decline in 2024 further suggests that the field remains active, but its development may be shaped by changing research priorities, target populations, and outcome domains.

**Figure 3:**
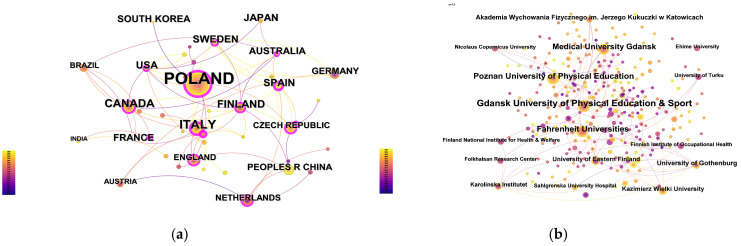
Country and institutional co-occurrence networks. (**a**) Country co-occurrence network; (**b**) institutional co-occurrence network. Node size represents publication frequency and links indicate co-occurrence relationships between countries or institutions. Frequency values for the top-ranked countries and institutions are summarized in Table 1 and Table 2, respectively.

Figure 3 shows the country and institutional collaboration networks, and the top-ranked nodes are summarized in Table 1 and Table 2. At the country level, Poland was the most prominent contributor, with the highest frequency and a high betweenness centrality value (*n* = 79, centrality = 0.33). Italy (*n* = 25, centrality = 0.23), Canada (*n* = 20, centrality = 0.20), and Finland (*n* = 16, centrality = 0.17) also showed relatively high frequencies and occupied visible positions in the network. Spain had a lower frequency than these countries (*n* = 13), but its centrality was also high (0.33), indicating that it may have acted as an important connector in the collaboration network. Overall, the country network suggests that research activity was concentrated around Poland and several European countries, while collaboration also extended to non-European contributors, including Canada, the USA, Australia, Japan, South Korea, and China.

The institutional co-occurrence network is presented in Figure 3b and the top-ranked institutions are summarized in Table 2. The most frequent institutions were Gdansk University of Physical Education and Sport (*n* = 23), Poznan University of Physical Education (*n* = 16), Fahrenheit Universities (*n* = 16), and Medical University Gdansk (*n* = 15). Other frequently occurring institutions included the University of Gothenburg (*n* = 8), Akademia Wychowania Fizycznego im. Jerzego Kukuczki w Katowicach (*n* = 8), the University of Eastern Finland (*n* = 7), Kazimierz Wielki University (*n* = 7), Karolinska Institutet (*n* = 6), and the University of Turku (*n* = 5). Most institutions showed low betweenness centrality values, suggesting that institutional collaboration in this field was relatively dispersed and that stronger cross-institutional collaboration may be needed in future research.

### 3.2. Intellectual Base and Co-Citation Structure

**Figure 4:**
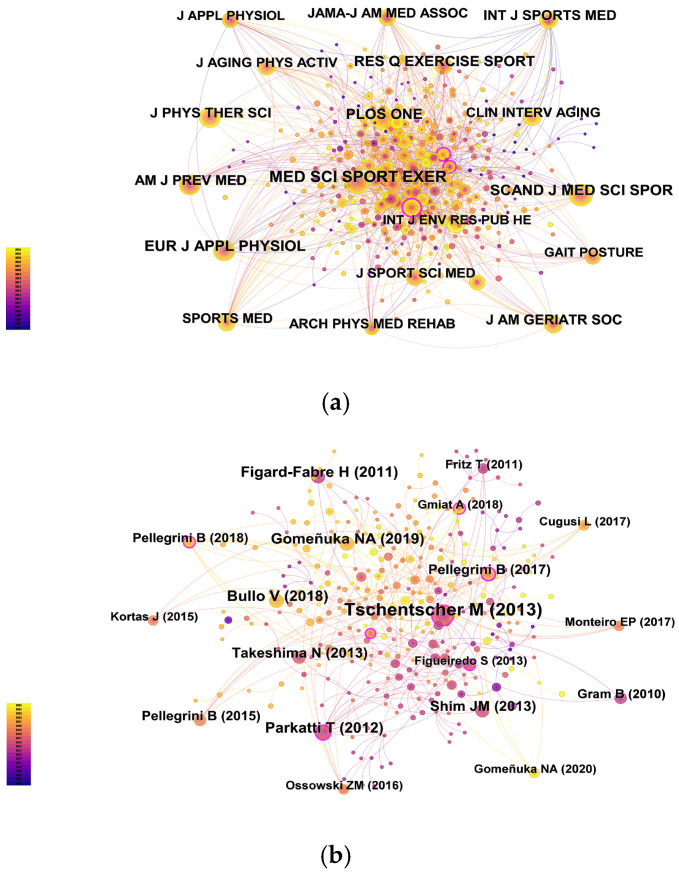
Journal and reference co-citation networks. (**a**) Journal co-citation network. (**b**) Reference co-citation network. Node size represents co-citation frequency, and links indicate co-citation relationships between nodes.

Figure 4a presents the journal and reference co-citation networks. The journal co-citation network comprised 416 nodes and 1082 links, with a density of 0.0125, whereas the reference co-citation network comprised 558 nodes and 560 links, with a density of 0.0036. Both networks showed a well-defined clustering structure (Q = 0.758; S = 0.9739). The journal co-citation network shows that the knowledge base of this field was mainly drawn from sport and exercise science, rehabilitation, gerontology, and clinical health research. The detailed ranking of co-cited journals is provided in Supplementary Table S1. The most frequently co-cited journals were Medicine & Science in Sports & Exercise (*n* = 146), European Journal of Applied Physiology (*n* = 105), Scandinavian Journal of Medicine & Science in Sports (*n* = 103), PLOS ONE (*n* = 87), and Research Quarterly for Exercise and Sport (*n* = 86). Other highly co-cited journals, including the American Journal of Preventive Medicine, International Journal of Sports Medicine, Journal of Physical Therapy Science, Journal of the American Geriatrics Society, and Clinical Interventions in Ageing, further indicate that this research area is supported by both exercise-related and health-oriented evidence sources.

The reference co-citation network (Figure 4b) identifies the publications that were most frequently cited together and therefore formed the core intellectual base of subsequent studies. As shown in Table 3, Tschentscher et al. (2013) was the most frequently co-cited reference (*n* = 34, centrality = 0.19), followed by Gomeñuka et al. (2019; *n* = 18), Bullo et al. (2018; *n* = 18), Figard-Fabre et al. (2011; *n* = 17), and Parkatti et al. (2012; *n* = 17). Pellegrini et al. (2017) had a lower co-citation frequency (*n* = 14), but showed the highest centrality among the top-ranked references (centrality = 0.28), suggesting that it occupied an important bridging position in the co-citation structure [23,24,25,26]. Taken together, these co-cited references indicate that the intellectual base of the field has developed around four connected strands: Nordic walking intervention evaluation, physiological and biomechanical response, gait and functional assessment, and rehabilitation- or clinically oriented applications.

### 3.3. Keyword Hotspots and Thematic Clustering

**Figure 5:**
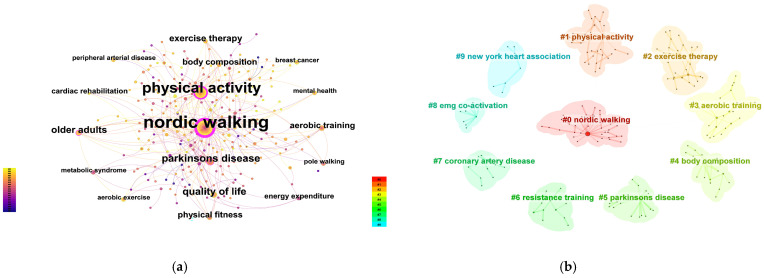
Keyword co-occurrence and clustering networks. (**a**) Keyword co-occurrence network. (**b**) Keyword clustering network. Node size represents keyword frequency and links indicate co-occurrence relationships between keywords.

The keyword network showed that the visible research vocabulary of the field was centered on Nordic walking, physical activity, exercise therapy, aerobic training, quality of life, physical fitness, and older adults. Disease- and rehabilitation-related terms, including Parkinson’s disease, cardiac rehabilitation, peripheral arterial disease, and breast cancer, also appeared in the network, suggesting that Nordic walking has been examined not only as a general physical activity modality but also in relation to functional health, rehabilitation, and specific clinical populations (Figure 5a).

The clustering results further organized these keywords into ten relatively well-defined groups. The largest clusters were #0 Nordic walking (size = 43, silhouette = 0.993, mean year = 2017), #1 physical activity (size = 31, silhouette = 0.983, mean year = 2020), #2 exercise therapy (size = 29, silhouette = 0.920, mean year = 2017), and #3 aerobic training (size = 25, silhouette = 0.975, mean year = 2018). The remaining clusters were #4 body composition, #5 Parkinson’s disease, #6 resistance training, #7 coronary artery disease, #8 EMG co-activation, and #9 New York Heart Association. These clusters indicate that the field has developed around both general exercise-related themes and more specific rehabilitation or disease-oriented applications (Figure 5b).

### 3.4. Evolution of Research Fronts over Time

**Figure 6:**
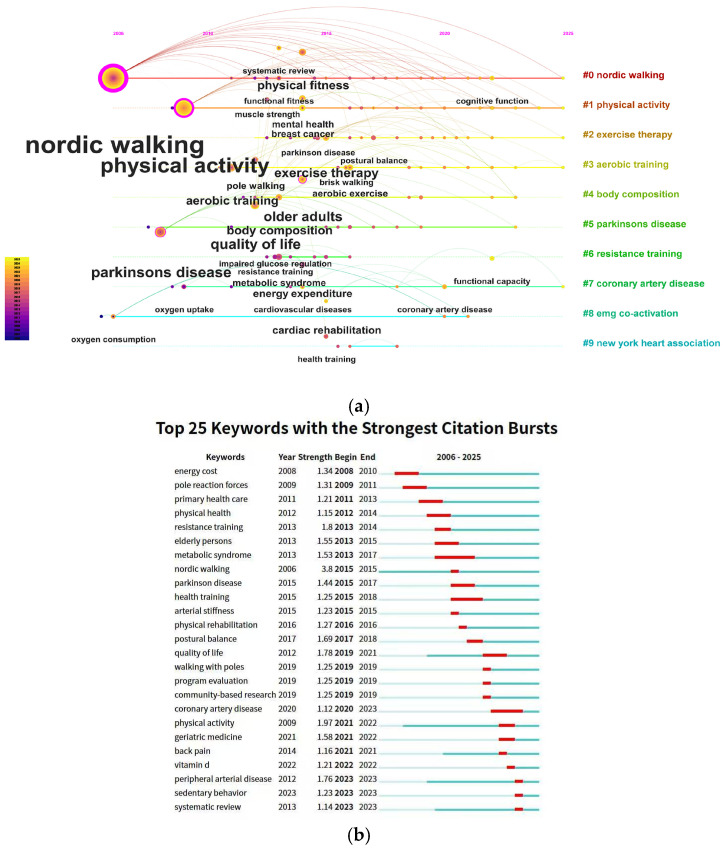
Temporal evolution and burst keywords of research fronts. (**a**) Timeline visualization of keyword clusters. (**b**) Top keywords with the strongest citation bursts. Red-green lines indicate the overall observation period, while red segments denote the years in which the corresponding keywords experienced strong citation bursts.

The timeline visualization indicates that the research fronts developed from general exercise-related and physiological topics toward more population- and health-oriented applications. Early terms such as energy expenditure, oxygen consumption, pole walking, and physical fitness suggest that initial studies focused on the physiological and functional characteristics of Nordic walking. Over time, the field expanded toward older adults, Parkinson’s disease, exercise therapy, aerobic training, body composition, and quality of life, indicating a growing interest in Nordic walking as an intervention strategy in ageing, rehabilitation, and chronic disease contexts (Figure 6a).

The burst keyword analysis further shows this temporal shift. Earlier bursts, including energy cost (2008–2010), pole reaction forces (2009–2011), primary health care (2011–2013), and physical health (2012–2014), were mainly related to physiological demand, movement characteristics, and general health promotion. Later bursts, such as metabolic syndrome, Parkinson’s disease, physical rehabilitation, and postural balance, reflected increasing attention to disease-related rehabilitation and functional outcomes. More recent bursts, including quality of life (2019–2021), coronary artery disease (2020–2023), physical activity (2021–2022), geriatric medicine (2021–2022), peripheral arterial disease (2023), sedentary behavior (2023), and systematic review (2023), suggest that current research has become more closely connected with clinical relevance, ageing-related health, lifestyle behavior, and evidence synthesis (Figure 6b).

### 3.5. LDA-Based Latent Topic Identification

**Figure 7:**
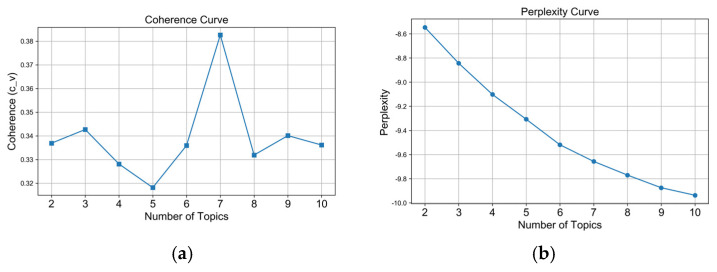
Coherence and perplexity plots for LDA topic-number selection. (**a**) C_v coherence score plot. (**b**) Perplexity plot.

To characterize the latent thematic structure of the corpus, LDA topic modeling was performed on the titles and abstracts of the included records. Candidate LDA models with K values ranging from 2 to 10 were evaluated using the C_v coherence score and the interpretability of the resulting topic keywords. As shown in Figure 7a, the coherence scores reached their highest value at K = 7. Figure 7b shows that perplexity continued to decrease as K increased, but the incremental reduction became smaller after K = 7. In addition, inspection of the topic keyword profiles indicated that the seven-topic solution produced interpretable and distinguishable topics. Therefore, K = 7 was selected as the final topic number for subsequent topic identification and temporal analysis.

**Figure 8:**
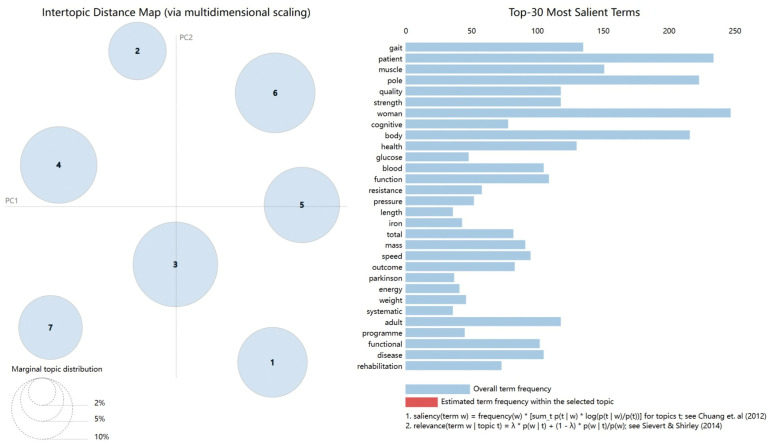
Intertopic distance map and salient terms of the LDA model. In the intertopic distance map, each circle corresponds to one LDA topic, and its size reflects the topic’s relative prevalence in the corpus. The spatial distance between circles represents semantic dissimilarity across topics. The bar chart displays the top salient terms across the corpus.

Based on the selected seven-topic solution, the LDA visualization revealed a latent semantic structure with several related but distinguishable thematic areas. As shown in Figure 8, the intertopic distance map showed that the seven topics were distributed across different regions of the semantic space, suggesting that the corpus was not dominated by a single homogeneous topic. The salient terms across the corpus, including *gait*, *patient*, *muscle*, *pole*, *quality*, *strength*, *cognitive*, *body composition*, *resistance*, *pressure*, and *rehabilitation*, indicate that the literature was broadly organized around the links between Nordic walking, physical function, rehabilitation, physiological adaptation, and health-related outcomes across different populations. Topic labels were assigned according to high-probability terms, topic-specific relevant terms, and semantic coherence within each topic. Topic-specific relevant-term distributions are provided in Supplementary Figure S1.

Topic 1 was labeled “Health outcomes and evaluative evidence.” This topic represents a broad evaluative cluster centered on the health-related effects and evidence base of Nordic walking. Its high-probability terms included quality, health, outcome, risk, therapy, treatment, disease, fall, musculoskeletal, systematic, database, randomized, report, and assessment. Together, these terms indicate studies that assessed Nordic walking in relation to quality of life, disease or fall risk, musculoskeletal health, and general intervention outcomes. At the same time, terms such as systematic, database, randomized, and assessment suggest that this topic also included work concerned with evidence synthesis and outcome evaluation. Compared with topics focused on a specific mechanism or disease group, Topic 1 therefore reflects a more general evidence-oriented strand of the literature, where Nordic walking is evaluated as a health-promoting intervention across broader populations and outcomes.

Topic 2 was labeled “Metabolic health and cardiovascular risk indicators.” This topic was defined by terms such as glucose, blood, pressure, lipid, overweight, metabolic, cholesterol, systolic, anthropometric, weight, body, mass, diet, patient, and rehabilitation. These terms indicate a focus on quantifiable cardiometabolic and anthropometric outcomes, including blood glucose, blood pressure, lipid profile, cholesterol level, body weight, and body composition-related indicators. In contrast to the broader evaluative orientation of Topic 1, Topic 2 reflects a more measurement-based strand of the literature, where Nordic walking is examined in relation to chronic disease risk factors and metabolic health. Terms such as program, week, control, and rehabilitation further suggest that this topic was often embedded in structured intervention or rehabilitation settings, where changes in health indicators were evaluated over a defined training period.

Topic 3 was labeled “Clinical rehabilitation and functional fitness improvement.” This topic was defined by terms such as patient, walking, test, rehabilitation, outcome, fitness, capacity, distance, functional, balance, score, cardiovascular, coronary, heart, pain, speed, and knee. These terms indicate a focus on rehabilitation-oriented applications of Nordic walking, with outcomes commonly related to walking distance, balance, exercise capacity, functional test scores, pain, and physical fitness. The appearance of cardiovascular, coronary, heart, and treadmill suggests that some studies also involved cardiac or cardiovascular rehabilitation settings. In contrast to Topic 2, which centered on measurable cardiometabolic indicators, Topic 3 reflects a more function-oriented strand of the literature, where Nordic walking is examined in relation to physical capacity, mobility, and practical rehabilitation outcomes.

Topic 4 was labeled “Exercise physiology and energy metabolism.” This topic was defined by terms such as pole, walking, body, energy, oxygen, rate, force, intensity, physiological, speed, heart, expenditure, metabolism, consumption, and response. These terms indicate a focus on the physiological and metabolic demands of Nordic walking, including energy expenditure, oxygen uptake, exercise intensity, heart-related responses, movement speed, and force production. In contrast to Topic 2, which centered on cardiometabolic risk indicators, Topic 4 was more closely related to the acute physiological load and exercise-response characteristics of Nordic walking. The appearance of pole, limb, force, and parameter further suggests that movement mechanics and pole-assisted locomotion were also important elements of this topic.

Topic 5 was labeled “Cognitive function and health maintenance.” This topic was characterized by terms such as walking, Nordic, cognitive, function, test, adult, week, aerobic, elderly, functional, capacity, session, control, health, healthy, ability, functioning, vitamin, serum, and maintain. These terms suggest that this topic focused on the role of Nordic walking in cognitive function and the maintenance of functional health, particularly among older or generally healthy adults. In contrast to the rehabilitation-oriented focus of Topic 3, Topic 5 was more closely related to preventive health, regular aerobic activity, cognitive performance, and functional capacity. The appearance of blood, serum, vitamin, and beta further suggests that some studies also considered biological or nutritional markers alongside cognitive or functional outcomes.

Topic 6 was labeled “Body composition, muscle strength, and nutritional supplementation.” This topic was defined by terms such as woman, muscle, body, strength, mass, composition, functional, elderly, vitamin, upper, performance, test, supplementation, postmenopausal, extremity, and flexibility. These terms indicate a focus on body composition, muscle strength, functional performance, and flexibility, particularly in women, older adults, or postmenopausal populations. In contrast to Topic 2, which centered on cardiometabolic risk indicators, Topic 6 was more closely related to musculoskeletal function and body structure. The appearance of vitamin and supplementation further suggests that some studies also considered nutritional or supplementation-related factors alongside Nordic walking when examining physical function and health maintenance.

Topic 7 was labeled “Gait parameters and Parkinson’s disease rehabilitation.” This topic was characterized by terms such as gait, pole, walking, speed, length, Parkinson, parameter, disease, step, pattern, motor, stride, motion, phase, posture, cycle, velocity, and movement. These terms suggest that the topic focused on gait characteristics and motor-control outcomes, including walking speed, step or stride length, gait pattern, movement phase, posture, and velocity. The appearance of Parkinson, motor, and disease further indicates a close connection with rehabilitation studies involving Parkinson’s disease or movement-related conditions. Compared with Topic 3, which captured broader functional rehabilitation outcomes, Topic 7 was more specifically concerned with walking structure, locomotor control, and neuromotor rehabilitation.

Across the seven topics, broad terms related to walking, health, function, and the body appeared in more than one topic, whereas the dominant analytical emphasis of each topic differed. Topic 1 covered broad health-outcome evaluation and evidence synthesis; Topic 2 focused on cardiometabolic risk and anthropometric indicators; Topic 3 addressed functional and clinical rehabilitation; Topic 4 examined physiological responses and movement mechanics; Topic 5 concerned cognitive health and preventive functional maintenance; Topic 6 focused on body composition, musculoskeletal function, and supplementation-related factors; and Topic 7 addressed gait characteristics, locomotor control, and Parkinson’s disease-related rehabilitation. Collectively, these topics represented complementary thematic emphases spanning health-outcome evaluation, risk indicators, physiological mechanisms, functional rehabilitation, and population-specific applications.

### 3.6. Topic Strength and Temporal Dynamics

**Figure 9:**
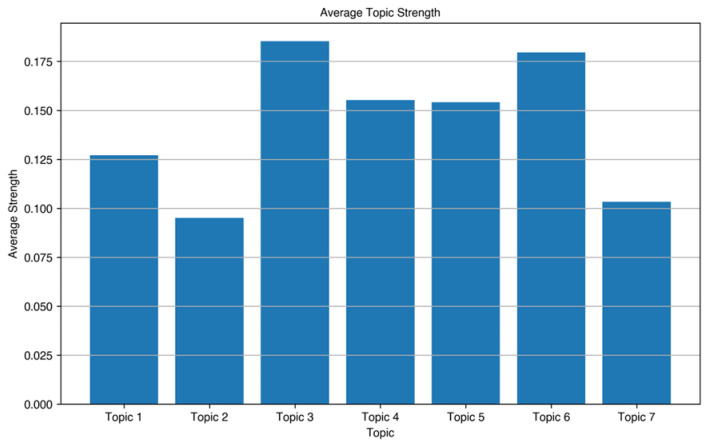
Average topic strength across the seven LDA topics.

Average topic strength was used to compare the relative weight of each latent topic across the corpus. The results show that thematic attention was not evenly distributed across the seven LDA topics (Figure 9). Topic 3 had the highest average strength (0.185), suggesting that clinical rehabilitation and functional fitness improvement represented the most prominent semantic focus. Topic 6 followed closely (0.180), indicating strong attention to body composition, muscle strength, and nutritional supplementation. Topics 4 and 5 showed comparable intermediate strengths (0.155 and 0.154, respectively), reflecting sustained interest in exercise physiology, energy metabolism, cognitive function, and health maintenance. Topic 1 had a moderate strength value (0.127), whereas Topics 7 and 2 showed lower values (0.103 and 0.095, respectively), suggesting that gait-oriented Parkinson’s disease rehabilitation and cardiometabolic risk indicators were more focused but less dominant thematic streams.

The yearly topic-strength results show temporal variation in the relative prominence of the seven LDA topics. In the early years, Topic 3 and Topic 4 showed relatively higher values in several individual years. These early-year values are interpreted as initial thematic signals rather than stable temporal peaks. Topic 3 was associated with clinical rehabilitation and functional fitness improvement, whereas Topic 4 reflected attention to exercise physiology, energy expenditure, oxygen uptake, and exercise-response characteristics.

From the middle period onward, the thematic structure became more diversified. Topic 2 increased during 2014–2016, indicating a temporary rise in attention to metabolic health and cardiovascular risk indicators. Topic 5 showed noticeable values in 2008, 2012, and again during 2019–2022, suggesting continued interest in cognitive function and health maintenance. Topic 6 became more visible after 2018 and reached its highest value in 2024 (0.36), indicating growing attention to body composition, muscle strength, and nutritional supplementation. In the most recent years, Topic 1 increased from 2022 onward and peaked in 2025 (0.28), while Topic 7 also rose in 2025 (0.16), suggesting renewed attention to health-outcome evaluation and gait- or Parkinson’s disease-related rehabilitation.

**Figure 10:**
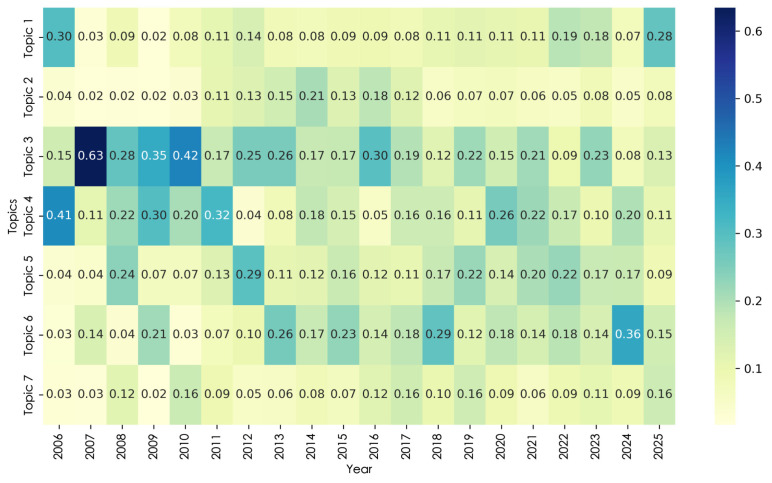
Topic Strength Heatmap Over Time (2006–2025).

**Figure 11:**
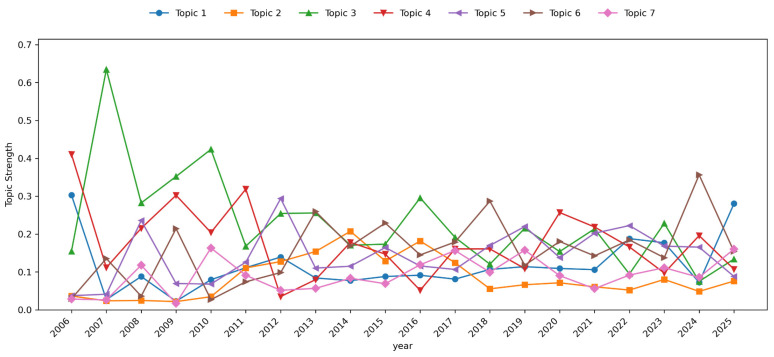
Topic Strength Over Time.

Overall, the temporal topic-strength patterns suggest that Nordic walking research did not develop along a single linear trajectory. Early studies were more strongly centered on functional rehabilitation and physiological response, whereas later studies showed a broader distribution across metabolic health, cognitive function, body composition, health-outcome evaluation, and gait-related rehabilitation (Figure 10 and Figure 11).

**Figure 12:**
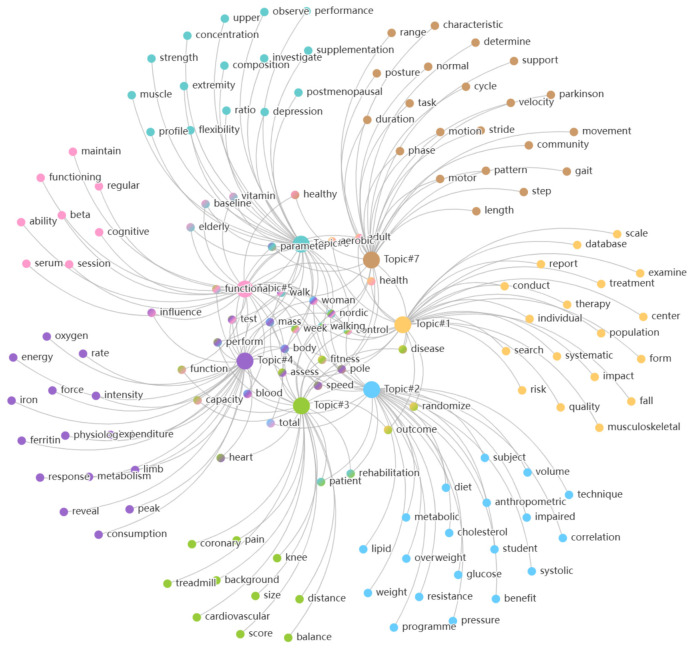
Topic–keyword relationship network derived from the LDA model.

As illustrated in Figure 12, the topic–keyword network was constructed to show how the seven LDA topics were connected through their associated terms. The network does not present the topics as isolated categories. Instead, several shared or centrally located terms, including walking, Nordic, health, function, body, week, control, fitness, woman, and elderly, form a semantic core that links the different topic branches. These terms indicate that most topics were grounded in a common intervention context: Nordic walking as a structured exercise activity evaluated through health, functional, and population-related outcomes.

Around this shared core, the network separates into several more specific branches. Topic 1 extends toward evaluative and health-outcome terms such as quality, systematic, database, risk, fall, musculoskeletal, and impact, reflecting the evidence-evaluation strand of the corpus. Topic 2 is connected with glucose, pressure, lipid, cholesterol, overweight, anthropometric, and systolic, showing a clear cardiometabolic and risk-indicator branch. Topic 3 is positioned close to terms such as rehabilitation, capacity, balance, distance, pain, knee, and score, indicating a clinically oriented functional-rehabilitation branch. Topic 4 is grouped around oxygen, energy, force, intensity, expenditure, consumption, and metabolism, representing the physiological-response and energy-metabolism branch. The upper and left parts of the network further show how the remaining topics extend this structure. Topic 5 is linked with cognitive, ability, functioning, regular, serum, vitamin, and maintain, suggesting a branch concerned with cognitive function and health maintenance. Topic 6 is associated with muscle, strength, composition, flexibility, postmenopausal, supplementation, and extremity, indicating a body-composition and musculoskeletal-function branch, particularly in women or older populations. Topic 7 forms a gait- and neuromotor-oriented branch through terms such as gait, Parkinson, motor, stride, velocity, posture, phase, cycle, and movement. This pattern indicates that the LDA topics were not isolated categories but were connected through shared terms related to Nordic walking, functional assessment, health outcomes, and rehabilitation contexts.

## 4. Discussion

### 4.1. Main Findings

This study mapped the research landscape of Nordic walking in ageing-related populations from 2006 to 2025 using CiteSpace and LDA topic modeling. The retrieved literature was not organized around a single research direction. Instead, it covered several connected areas, including exercise physiology, physical activity, functional health, metabolic and cardiovascular risk-related outcomes, gait analysis, and rehabilitation-related applications. This interdisciplinary structure suggests that Nordic walking has developed beyond a technical variation of walking or an isolated exercise intervention. It has increasingly become a research context for examining relationships among exercise characteristics, physiological responses, functional performance, rehabilitation, and ageing-related health outcomes.

The concentration of publications in several European countries, together with relatively dispersed institutional connections, indicates that the field has developed through a number of active regional research groups rather than through a highly integrated international network. Reporting these country-level patterns provides a broader overview of where Nordic walking research has been concentrated and may help identify opportunities for wider international collaboration. These patterns describe research activity and collaboration within the field, rather than differences in the health effects of Nordic walking across locations. Greater multicentre and international collaboration may support methodological comparability in future studies [27,28,29].

The principal contribution of the present study is not simply the identification of seven topics, but their integration into a layered interpretation of the field. The literature can be understood as involving four connected levels: (1) exercise and biomechanical characteristics; (2) physiological and physical adaptations; (3) functional performance; and (4) clinical, cognitive, quality-of-life, and broader ageing-related health outcomes. This structure suggests that the field has developed from characterizing Nordic walking as an exercise modality toward investigating how its mechanisms and physical adaptations may relate to meaningful functional and health outcomes [30]. These levels should not be interpreted as a causal pathway validated by the present analysis. Rather, they provide a conceptual structure for explaining how the major themes within the WoSCC-indexed corpus are connected. The academic contribution therefore lies in clarifying the internal organization of the field and identifying where relationships among exercise mechanisms, physical adaptations, functional performance, populations, and health outcomes remain insufficiently examined.

### 4.2. Shift in Research Focus Toward Applied Health Contexts

The early concentration on exercise physiology and biomechanics reflects a foundational stage in Nordic walking research. Because pole-assisted walking involves active upper-body engagement and altered locomotor coordination compared with conventional walking, early studies needed to establish its distinctive physiological and biomechanical profile. Research on energy expenditure, oxygen uptake, exercise intensity, pole forces, and movement coordination therefore provided the basis for later work on functional and clinical applications [31].

As this profile became clearer, research attention shifted from defining the exercise mode to examining its relevance across ageing-related and clinical contexts. Nordic walking retains the familiar movement pattern of walking while allowing adjustments in speed, terrain, pole technique, session duration, and training progression. Its relatively low equipment requirements and active upper-body involvement have supported its use in supervised rehabilitation, organized group exercise, and community-based programs for people with different levels of functional capacity [32,33,34].

This development can be interpreted as a movement from mechanism to function and application. In ageing-related populations, physiological adaptation, musculoskeletal function, mobility, cognition, and clinical status are interconnected rather than isolated concerns. The expansion of the literature toward functional capacity, rehabilitation, chronic disease-related applications, cognitive health, and broader health outcomes therefore reflects the need to examine Nordic walking within this multidimensional context [35,36]. Future research should further connect physiological and gait-related measures with functionally and clinically meaningful outcomes through population-specific study designs.

### 4.3. LDA Topic Structure and Thematic Relationships

The LDA results complement the CiteSpace analysis by explaining how the visible research hotspots are connected at the semantic level. Instead of treating the seven topics as separate categories, the topic structure suggests that Nordic walking research is organized around a common intervention context, with several extensions toward health evaluation, rehabilitation, physiological mechanisms, and population-specific applications.

One important relationship is the connection between general health-outcome evaluation and measurable clinical risk indicators. Topic 1 and Topic 2 together suggest that Nordic walking has been evaluated through two complementary pathways. The first pathway focuses on broad health outcomes, such as quality of life, fall risk, musculoskeletal health, and evidence synthesis. The second pathway focuses on more specific cardiometabolic indicators. This relationship suggests that Nordic walking is not only discussed as a general health-promoting activity, but also as an intervention that can be examined through measurable risk-related outcomes. However, these two evaluation pathways appear to remain only loosely connected in the existing topic structure. Future studies could further examine how changes in cardiometabolic indicators are related to broader health outcomes such as quality of life, functional independence, and fall-related risk [37,38].

A second relationship appears between functional rehabilitation and gait-oriented recovery. Topic 3 and Topic 7 are closely related because both concern rehabilitation and physical function, but they differ in emphasis. Topic 3 reflects a broader functional-rehabilitation direction, including walking capacity, balance, pain, exercise capacity, and clinical rehabilitation outcomes. Topic 7 is more specific to gait parameters, locomotor control, Parkinson’s disease, motor function, posture, and movement patterns. This distinction indicates that Nordic walking rehabilitation research has developed at two levels: a general functional-recovery level and a more detailed gait and neuromotor-control level. The potential gap is that functional outcomes and gait-mechanism outcomes are often discussed as separate points. More integrated studies are needed to clarify whether improvements in gait parameters or motor control translate into clinically meaningful gains in balance, mobility, pain reduction, or daily functional performance [39].

A third relationship links physiological mechanisms with population-specific applications. Topic 4 provides the physiological basis of Nordic walking, including energy expenditure, oxygen uptake, exercise intensity, force, and exercise response. Topic 5 and Topic 6 extend this mechanism-oriented perspective toward specific health and population contexts, including cognitive function, health maintenance, body composition, muscle strength, nutritional supplementation, and older female populations. This pattern indicates that research attention has expanded from mechanism-oriented studies toward broader functional, clinical, and population-specific applications. Nevertheless, the current topic structure suggests that mechanism-oriented studies and population-specific intervention studies are not yet fully integrated. Future research could better connect physiological load, muscle function, cognitive outcomes, and body-composition changes within specific groups such as older adults, women, or patients with chronic conditions [14,40]. Together, these relationships suggest that the next stage of Nordic walking research should place greater emphasis on linking mechanisms, outcomes, and target populations rather than examining them as separate research directions.

### 4.4. Future Research Directions

Based on these findings, several areas appear particularly important for future Nordic walking research, including intervention standardization, mechanism–outcome integration, population-specific protocols, and stronger research collaboration.

First, future studies should strengthen the design and reporting of Nordic walking interventions. As Nordic walking is applied to different populations and health conditions, differences in intervention duration, weekly frequency, session intensity, supervision, pole technique, progression, and comparator conditions become more important. Comparative research on Nordic walking and ordinary walking in ageing-related populations may provide further insight into the development of the field. Such work could help clarify which outcomes are shared across walking-based exercise and which warrant more specific attention in relation to pole-assisted walking. More standardized reporting of training dose, adherence, progression, technique instruction, comparator conditions, and adverse events would improve the comparability and cumulative value of future evidence.

Second, future research should better connect physiological mechanisms with functional and clinical outcomes. The topic structure shows related but still partly separated areas involving exercise physiology, energy metabolism, rehabilitation, and gait parameters. Future studies could examine how changes in oxygen uptake, energy expenditure, muscle activation, force production, or gait parameters are associated with improvements in balance, walking capacity, pain, quality of life, or disease-specific function. This would help move the field from parallel outcome reporting toward clearer explanations of how Nordic walking may produce health-related changes.

Third, more population-specific research is needed. The LDA topics and temporal findings indicate growing attention to older adults, women, postmenopausal populations, patients with Parkinson’s disease, cardiometabolic risk, coronary artery disease, and peripheral arterial disease. These groups may differ in baseline function, safety considerations, training tolerance, and outcome priorities. Future studies should therefore develop tailored Nordic walking protocols and outcome sets for different populations rather than treating Nordic walking as a uniform intervention.

Fourth, the relatively dispersed institutional collaboration network suggests a need for stronger cross-institutional and international collaboration. Multi-center studies, shared intervention protocols, harmonized outcome measures, and open data practices could help reduce fragmentation and improve the cumulative value of Nordic walking evidence.

### 4.5. Strengths and Limitations

This study has several strengths. First, it combined CiteSpace-based science mapping with LDA topic modeling, allowing the analysis to capture both external knowledge structures and internal semantic themes. CiteSpace provided information on collaboration patterns, co-citation structures, keyword hotspots, and temporal research fronts, whereas LDA identified latent topic structures and topic-strength changes over time. Second, the study examined Nordic walking research from multiple angles, including geographical distribution, institutional collaboration, intellectual foundations, visible hotspots, emerging fronts, and latent thematic relationships. This multi-layered approach provides a more comprehensive overview than a single bibliometric or narrative method alone.

Several limitations should also be considered. First, the dataset was based on the selected bibliographic database and search strategy. Although this approach ensured relatively standardized metadata for bibliometric analysis, some relevant studies indexed in other databases or published in less visible sources may not have been included. In addition, although the corpus was retrieved using Topic-field terms associated with ageing-related Nordic walking research rather than participant age as an explicit eligibility criterion, some general-population-related terms emerged in the topic analysis. This does not alter the field-level focus of the present study on Nordic walking research relevant to ageing-related populations. In addition, although the corpus was retrieved using Topic-field terms associated with ageing-related Nordic walking research rather than participant age as an explicit eligibility criterion, some general-population-related terms emerged in the topic analysis. Physiology- or biomechanics-oriented records may also have entered the corpus and contributed to Topic 4 when their titles, abstracts, or indexed keywords linked them to ageing-related Nordic walking research. This does not alter the field-level focus of the present study on Nordic walking research relevant to ageing-related populations. Future studies could compare findings across multiple databases to further test the robustness of the research landscape identified here.

Second, the results of science mapping and topic modeling are influenced by methodological decisions, including keyword cleaning, parameter settings, text preprocessing, and topic labeling. To reduce arbitrary interpretation, the present study combined network-based results with LDA topic modeling and interpreted the topics according to high-probability terms, topic-specific relevant terms, and semantic coherence. Even so, alternative settings may produce slightly different topic structures.

Third, this study was designed to map the knowledge structure and thematic development of Nordic walking research rather than to evaluate the effectiveness of Nordic walking interventions. Therefore, the findings should be interpreted as a research-landscape and topic-structure analysis, not as direct evidence of intervention effects. Future systematic reviews, meta-analyses, and well-designed intervention studies are needed to examine the clinical and functional effects of Nordic walking in specific populations.

## 5. Conclusions

This study mapped Nordic walking research in ageing-related populations from 2006 to 2025 using CiteSpace-based bibliometric analysis and LDA topic modeling. Rather than showing only an expansion in the range of outcomes examined, the mapped literature suggests a change in how Nordic walking is framed. Early research mainly characterized pole-assisted walking through physiological, biomechanical, and gait-related features. Later work increasingly connected these features with functional capacity, rehabilitation, and ageing-related health concerns in specific populations. The field is therefore becoming organized not around isolated outcome domains, but around the relationship between movement characteristics, functional needs, and population-specific applications. This perspective moves Nordic walking beyond its description as a distinct walking modality and highlights its place at the intersection of movement science, rehabilitation, and healthy-ageing research. Future studies should build on this structure by defining the target population and functional problem more precisely, reporting pole technique and training dose in sufficient detail, and relating gait or physiological measures to predefined functional, clinical, or health-related outcomes. Such work can clarify which changes are common to walking-based activity and which may be more closely linked to the specific characteristics of Nordic walking, while improving the comparability of evidence across rehabilitation and healthy-ageing contexts.

## Figures and Tables

**Table 1:** Top 10 countries in the co-occurrence network.

Rank	Country	Frequency	Centrality	Year
1	Poland	79	0.33	2009
2	Italy	25	0.23	2010
3	Canada	20	0.20	2011
4	Finland	16	0.17	2007
5	Spain	13	0.33	2015
6	Japan	13	0.00	2013
7	Sweden	13	0.16	2010
8	USA	13	0.18	2012
9	France	11	0.03	2008
10	Australia	11	0.14	2011

Note: Frequency indicates the number of occurrences in the co-occurrence network. Centrality refers to betweenness centrality. Year indicates the first year in which the country/region appeared in the co-occurrence network.

**Table 2:** Top 10 institutions in the co-occurrence network.

Rank	Institution	Frequency	Centrality	Year
1	Gdansk University of Physical Education and Sport	23	0	2014
2	Poznan University of Physical Education	16	0	2015
3	Fahrenheit Universities	16	0	2015
4	Medical University Gdansk	15	0.01	2005
5	University of Gothenburg	8	0.01	2010
6	Akademia Wychowania Fizycznego im. Jerzego Kukuczki w Katowicach	8	0.01	2014
7	University of Eastern Finland	7	0.01	2013
8	Kazimierz Wielki University	7	0.01	2015
9	Karolinska Institutet	6	0.01	2011
10	University of Turku	5	0	2013

Note: Frequency indicates the number of occurrences in the co-occurrence network. Centrality refers to betweenness centrality. Year indicates the first year in which the country/region appeared in the co-occurrence network.

**Table 3:** Top 10 co-cited references.

Rank	Co-Cited Reference	Frequency	Centrality	Year
1	Tschentscher M, 2013, *American Journal of Preventive Medicine*	34	0.19	2013
2	Gomeñuka NA, 2019, *PLOS ONE*	18	0.03	2019
3	Bullo V, 2018, *Rejuvenation Research*	18	0.05	2018
4	Figard-Fabre H, 2011, *International Journal of Sports Medicine*	17	0.07	2011
5	Parkatti T, 2012, *Journal of Aging and Physical Activity*	17	0.12	2012
6	Shim JM, 2013, *Journal of Physical Therapy Science*	15	0.02	2013
7	Takeshima N, 2013, *Journal of Sports Science and Medicine*	15	0.04	2013
8	Pellegrini B, 2017, Gait & Posture	14	0.28	2017
9	Pellegrini B, 2015, *PLOS ONE*	13	0.03	2015
10	Pellegrini B, 2018, *PLOS ONE*	12	0.14	2018

## Data Availability

The data supporting the findings of this study are included in the article and Supplementary Materials.

## Supplementary Materials

The following supporting information can be downloaded at: https://www.mdpi.com/article/10.3390/healthcare14142061/s1, Figure S1: Topic-specific relevant-term distributions for the seven LDA topics; Table S1: Top 10 co-cited journals.

## Abbreviations

The following abbreviations are used in this manuscript:

LDA	Latent Dirichlet Allocation
WoS	Web of Science
LLR	Log-likelihood ratio
C_v	Coherence score

## References

[1] Patel R., Gallagher J.E. (2024). Healthy Ageing and Oral Health: Priority, Policy and Public Health. BDJ Open.

[2] Cruz-Jentoft A.J., Bahat G., Bauer J., Boirie Y., Bruyere O., Cederholm T., Cooper C., Landi F., Rolland Y., Sayer A.A. (2019). Sarcopenia: Revised European Consensus on Definition and Diagnosis. Age Ageing.

[3] Marconcin P., São Martinho E., Serpa J., Honório S., Loureiro V., Nascimento M.D.M., Flôres F., Santos V. (2025). Grip Strength, Fall Efficacy, and Balance Confidence as Associated Factors with Fall Risk in Middle-Aged and Older Adults Living in the Community. Appl. Sci..

[4] Van Der Ploeg H.P., Bull F.C. (2020). Invest in Physical Activity to Protect and Promote Health: The 2020 WHO Guidelines on Physical Activity and Sedentary Behaviour. Int. J. Behav. Nutr. Phys. Act..

[5] Calvani R., Cesari M., Marzetti E. (2024). Pacing Longevity: Serial Gait Speed Measurements and Survival in Older Adults. J. Nutr. Health Aging.

[6] Sherrington C., Fairhall N., Wallbank G., Tiedemann A., Michaleff Z.A., Howard K., Clemson L., Hopewell S., Lamb S. (2020). Exercise for Preventing Falls in Older People Living in the Community: An Abridged Cochrane Systematic Review. Br. J. Sports Med..

[7] Pellegrini B., Peyré-Tartaruga L.A., Zoppirolli C., Bortolan L., Bacchi E., Figard-Fabre H., Schena F. (2015). Exploring Muscle Activation during Nordic Walking: A Comparison between Conventional and Uphill Walking. PLoS ONE.

[8] Knappova V., Dorota K., Anna W., Kavalirova G., Zbigniew N., Gabryś T., Agata N.-L. (2025). Influence of Systematic Standard and Nordic Walking Training on Exercise Tolerance and Body Weight Components in Women over 55 Years of Age. Front. Sports Act. Living.

[9] Tschentscher M., Niederseer D., Niebauer J. (2013). Health Benefits of Nordic Walking. Am. J. Prev. Med..

[10] Skórkowska-Telichowska K., Kropielnicka K., Bulińska K., Pilch U., Woźniewski M., Szuba A., Jasiński R. (2016). Nordic Walking in the Second Half of Life. Aging Clin. Exp. Res..

[11] Gomeñuka N.A., Oliveira H.B., Silva E.S., Costa R.R., Kanitz A.C., Liedtke G.V., Schuch F.B., Peyré-Tartaruga L.A. (2019). Effects of Nordic Walking Training on Quality of Life, Balance and Functional Mobility in Elderly: A Randomized Clinical Trial. PLoS ONE.

[12] Carvalho A., Palma V.C., Tomás M.T. (2025). Exercise Prescription for Frail Older Adults: Impact on Handgrip Strength and Gait Speed—A Systematic Review. Phys. Occup. Ther. Geriatr..

[13] Sánchez-Basallote M., Monge-Pereira E., de Oliveira I.M., Mollinedo-Cardalda I. (2026). Efectos de la marcha nórdica en las personas con enfermedad de Parkinson: Revisión de revisiones sistemáticas. Rehabilitación.

[14] Li H., Zhu K., Gan J., Wang Z., Gao Z., Liu L., Guo X., Niu J. (2025). The Effects of Nordic Walking on Cognitive Function in Older Adults: A Systematic Review and Meta-Analysis. Front. Aging Neurosci..

[15] Takeshima N., Islam M.M., Rogers M.E., Rogers N.L., Koizumi D., Kitabayashi Y., Imai A., Naruse A. (2013). Effects of Nordic Walking Compared to Conventional Walking and Band-Based Resistance Exercise on Fitness in Older Adults. J. Sports Sci. Med..

[16] Kocur P., Wiernicka M., Wilski M., Kaminska E., Furmaniuk L., Maslowska M.F., Lewandowski J. (2015). Does Nordic Walking Improves the Postural Control and Gait Parameters of Women between the Age 65 and 74: A Randomized Trial. J. Phys. Ther. Sci..

[17] Krumpoch S., Lindemann U., Rappl A., Becker C., Sieber C.C., Freiberger E. (2021). The Effect of Different Test Protocols and Walking Distances on Gait Speed in Older Persons. Aging Clin. Exp. Res..

[18] Gil A.W.O., Oliveira M.R., Coelho V.A., Carvalho C.E., Teixeira D.C., Silva R.A.D. (2011). Relationship between Force Platform and Two Functional Tests for Measuring Balance in the Elderly. Rev. Bras. Fisioter..

[19] Chen C. (2018). Visualizing and Exploring Scientific Literature with CiteSpace: An Introduction. Proceedings of the 2018 Conference on Human Information Interaction & Retrieval—CHIIR ’18, New Brunswick, NJ, USA, 11–15 March 2018.

[20] Chen C. (2017). Science Mapping: A Systematic Review of the Literature. J. Data Inf. Sci..

[21] Blei D., Ng A., Jordan M. (2003). Latent Dirichlet Allocation. J. Mach. Learn. Res..

[22] Roeder M., Both A., Hinneburg A. (2015). Exploring the Space of Topic Coherence Measures. WSDM ’15: Proceedings of the Eighth ACM International Conference on Web Search and Data Mining.

[23] Figard-Fabre H., Fabre N., Leonardi A., Schena F. (2011). Efficacy of Nordic Walking in Obesity Management. Int. J. Sports Med..

[24] Parkatti T., Perttunen J., Wacker P. (2012). Improvements in Functional Capacity from Nordic Walking: A Randomized Controlled Trial Among Older Adults. J. Aging Phys. Act..

[25] Pellegrini B., Peyre-Tartaruga L.A., Zoppirolli C., Bortolan L., Savoldelli A., Minetti A.E., Schena F. (2017). Mechanical Energy Patterns in Nordic Walking: Comparisons with Conventional Walking. Gait Posture.

[26] Bullo V., Gobbo S., Vendramin B., Duregon F., Cugusi L., Di Blasio A., Bocalini D.S., Zaccaria M., Bergamin M., Ermolao A. (2018). Nordic Walking Can Be Incorporated in the Exercise Prescription to Increase Aerobic Capacity, Strength, and Quality of Life for Elderly: A Systematic Review and Meta-Analysis. Rejuvenation Res..

[27] Bulinska K., Kropielnicka K., Jasinski T., Wojcieszczyk-Latos J., Pilch U., Dabrowska G., Skorkowska-Telichowska K., Kalka D., Zywar K., Paszkowski R. (2016). Nordic Pole Walking Improves Walking Capacity in Patients with Intermittent Claudication: A Randomized Controlled Trial. Disabil. Rehabil..

[28] Mieszkowski J., Niespodziński B., Kochanowicz A., Gmiat A., Prusik K., Prusik K., Kortas J., Ziemann E., Antosiewicz J. (2018). The Effect of Nordic Walking Training Combined with Vitamin D Supplementation on Postural Control and Muscle Strength in Elderly People—A Randomized Controlled Trial. Int. J. Environ. Res. Public Health.

[29] Song M.-S., Yoo Y.-K., Choi C.-H., Kim N.-C. (2013). Effects of Nordic Walking on Body Composition, Muscle Strength, and Lipid Profile in Elderly Women. Asian Nurs. Res..

[30] Nemoto Y., Sakurai R., Ogawa S., Maruo K., Fujiwara Y. (2021). Effects of an Unsupervised Nordic Walking Intervention on Cognitive and Physical Function among Older Women Engaging in Volunteer Activity. J. Exerc. Sci. Fit..

[31] Schiffer T., Knicker A., Dannoehl R., Strueder H.K. (2009). Energy Cost and Pole Forces during Nordic Walking under Different Surface Conditions. Med. Sci. Sports Exerc..

[32] De Santis K.K., Kaplan I. (2020). The Motor and the Non-Motor Outcomes of Nordic Walking in Parkinson’s Disease: A Systematic Review. J. Bodyw. Mov. Ther..

[33] Salse-Batan J., Sanchez-Lastra M.A., Suarez-Iglesias D., Varela S., Ayan C. (2022). Effects of Nordic Walking in People with Parkinson’s Disease: A Systematic Review and Meta-Analysis. Health Soc. Care Community.

[34] Vilanova-Pereira M., Barral-Fernández M., Labata-Lezaun N., Llurda-Almuzara L., Pérez-Bellmunt A., Jácome C., Lista-Paz A. (2025). Effects of Nordic Walking in People with Respiratory Diseases: A Systematic Review and Meta-Analysis. J. Rehabil. Med..

[35] Harro C.C., Shoemaker M.J., Coatney C.M., Lentine V.E., Lieffers L.R., Quigley J.J., Rollins S.G., Stewart J.D., Hall J., Khoo S.K. (2022). Effects of Nordic Walking Exercise on Gait, Motor/Non-Motor Symptoms, and Serum Brain-Derived Neurotrophic Factor in Individuals with Parkinson’s Disease. Front. Rehabilit. Sci..

[36] Liu J., Kim J.-H. (2025). The Effects of Nordic Walking on the Cardiovascular Risk Factors in Older Adults: A Systematic Review and Meta-Analysis. Arch. Gerontol. Geriatr..

[37] Fritz T., Caidahl K., Osler M., Östenson C.G., Zierath J.R., Wändell P. (2011). Effects of Nordic Walking on Health-related Quality of Life in Overweight Individuals with Type 2 Diabetes Mellitus, Impaired or Normal Glucose Tolerance. Diabet. Med..

[38] Della Guardia L., Pellino V.C., Filipas L., Bonato M., Gallo G., Lovecchio N., Vandoni M., Codella R. (2023). Nordic Walking Improves Cardiometabolic Parameters, Fitness Performance, and Quality of Life in Older Adults with Type 2 Diabetes. Endocr. Pract..

[39] Espinoza-Araneda J., Caparros-Manosalva C., Da Cunha M., Marzuca-Nassr G.N., Fritz-Silva N., Pagnussat A.S. (2024). Nordic Walking and Arm Swing Asymmetry in People with Parkinson’s Disease: Protocol for a Randomised Clinical Trial. BMJ Open Sports Exerc. Med..

[40] Morano T., Lancia F., Di Marco A., Viscioni G., Bucci I., Grossi S., Pellegrino R., Cugusi L., Grassadonia A., Manca A. (2024). Flexibility and Strength Effects of Adapted Nordic Walking and Myofascial Exercises Practice in Breast Cancer Survivors and Analysis of Differences. Healthcare.

