# Supplementary material for: Mapping Nordic Walking Research in Ageing-Related Populations: A Bibliometric and Topic Modeling Analysis (2006–2025)
Source: Healthcare (Basel). 2026 Jul 9;14(14):2061. doi: 10.3390/healthcare14142061 (PMC13409863; doi:10.3390/healthcare14142061)
Supplement: Supplementary file 1 [file healthcare-14-02061-s001.zip › Supplementary Materials.pdf]

Supplementary Table S1. Top 10 co-cited journals

Supplementary Table s1. Top 10 co-cited journals.

Rank	Journal	Frequency	Centrality	Year
1	Medicine & Science in Sports & Exercise	146	0.01	2006
2	European Journal of Applied Physiology	105	0.10	2006
3	Scandinavian Journal of Medicine & Science in Sports	103	0.07	2007
4	PLOS ONE	87	0.04	2014
5	Research Quarterly for Exercise and Sport	86	0.05	2006
6	American Journal of Preventive Medicine	76	0.09	2008
7	International Journal of Sports Medicine	72	0.08	2006
8	Journal of Physical Therapy Science	72	0.02	2015
9	Journal of the American Geriatrics Society	70	0.05	2013
10	Clinical Interventions in Aging	64	0.06	2013

Note. Frequency indicates the co-citation frequency of each journal. Centrality refers to betweenness centrality in the journal co-citation network. Year indicates the first year in which the journal appeared in the network.

Supplementary Figure S1. Topic-specific relevant-term distributions for the seven LDA topics.

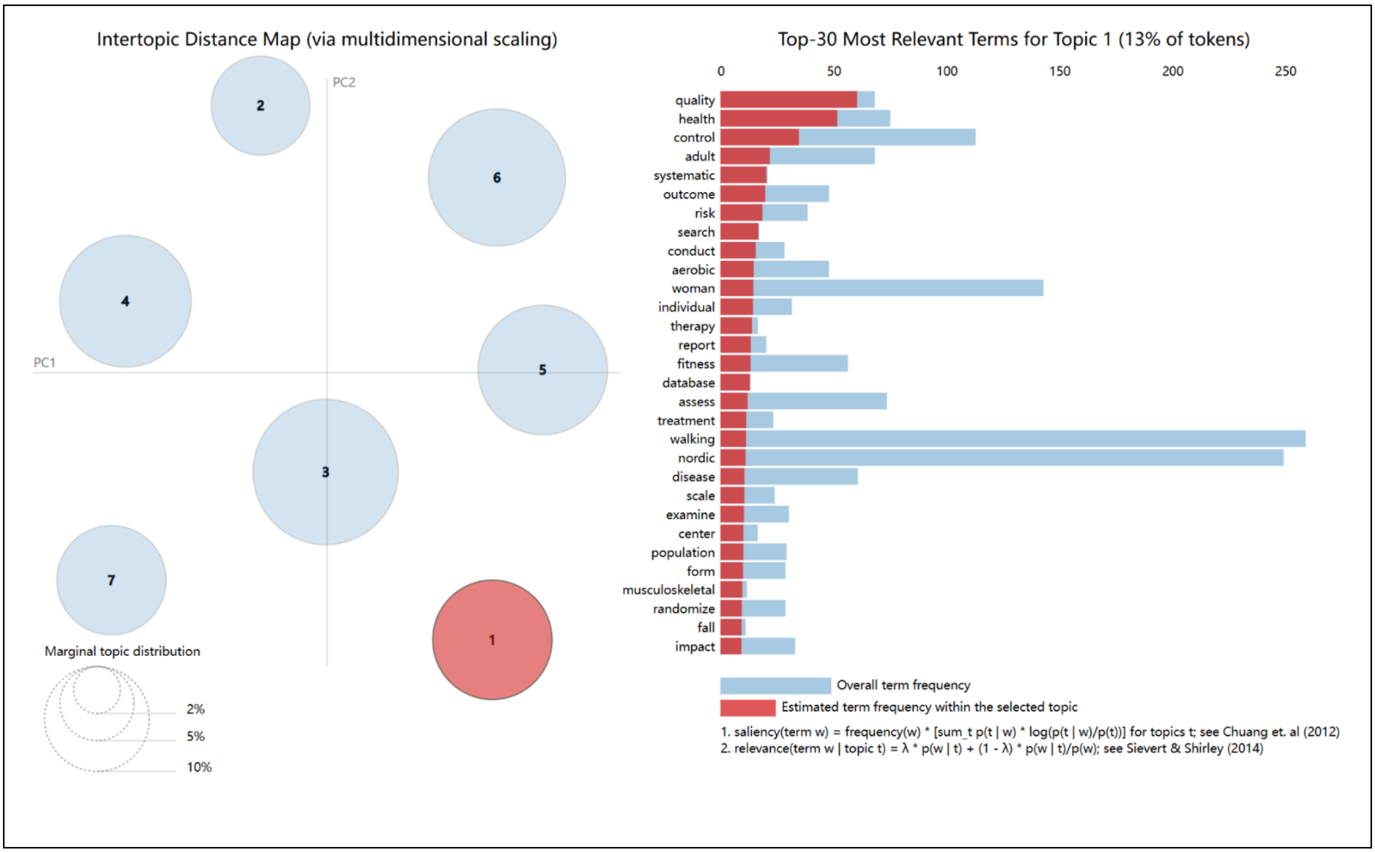

Supplementary Figure S1A. Topic 1: Health outcomes and evaluative evidence.

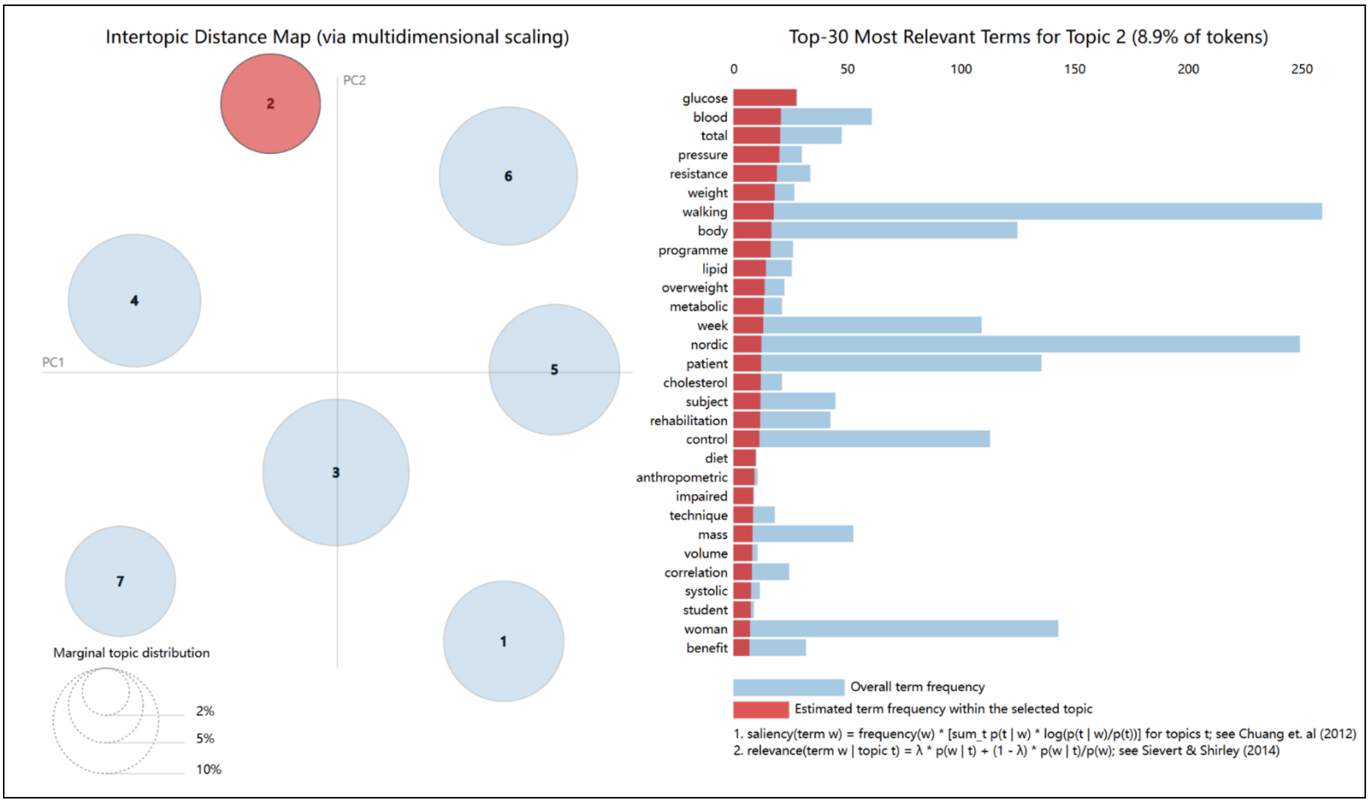

Supplementary Figure S1B. Topic 2: Metabolic health and cardiovascular risk indicators.

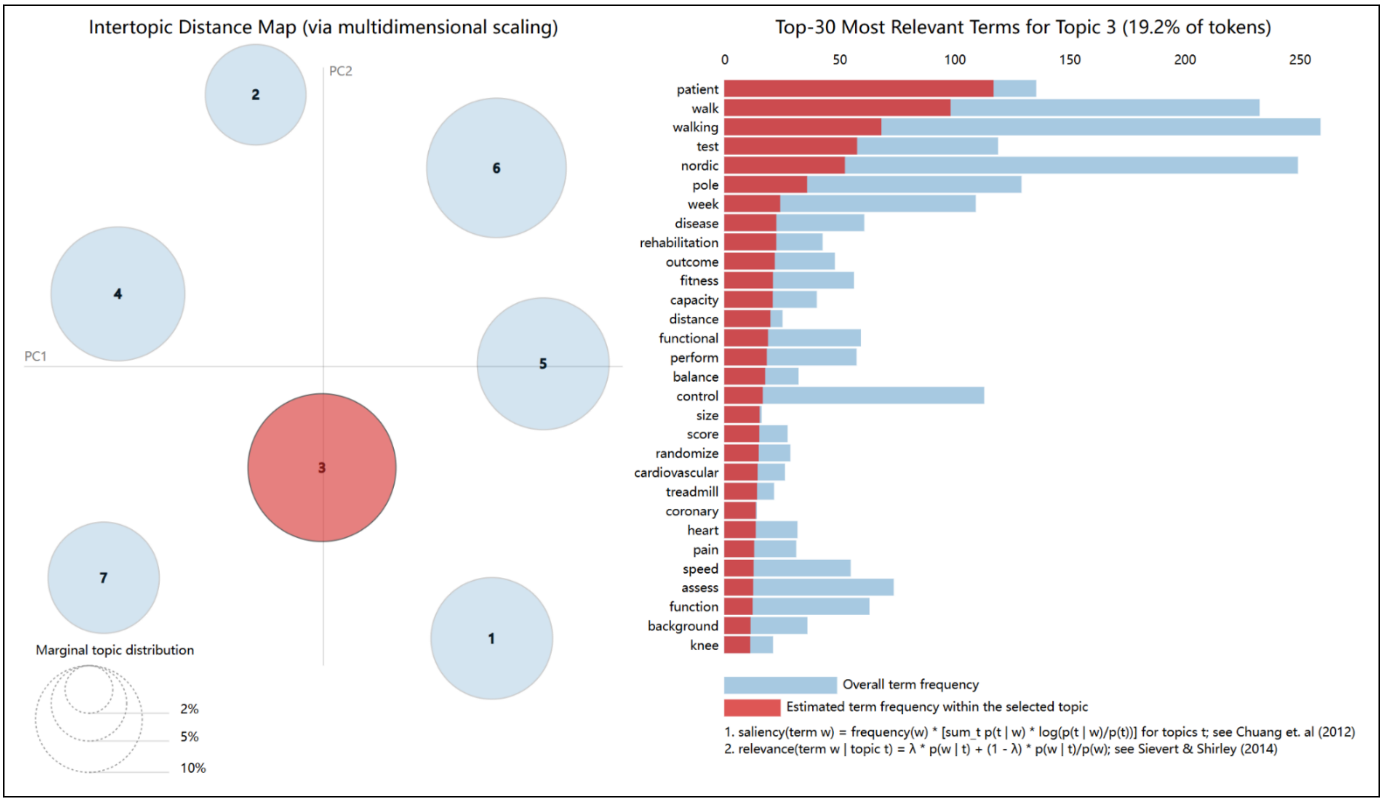

Supplementary Figure S1C. Topic 3: Clinical rehabilitation and functional fitness improvement.

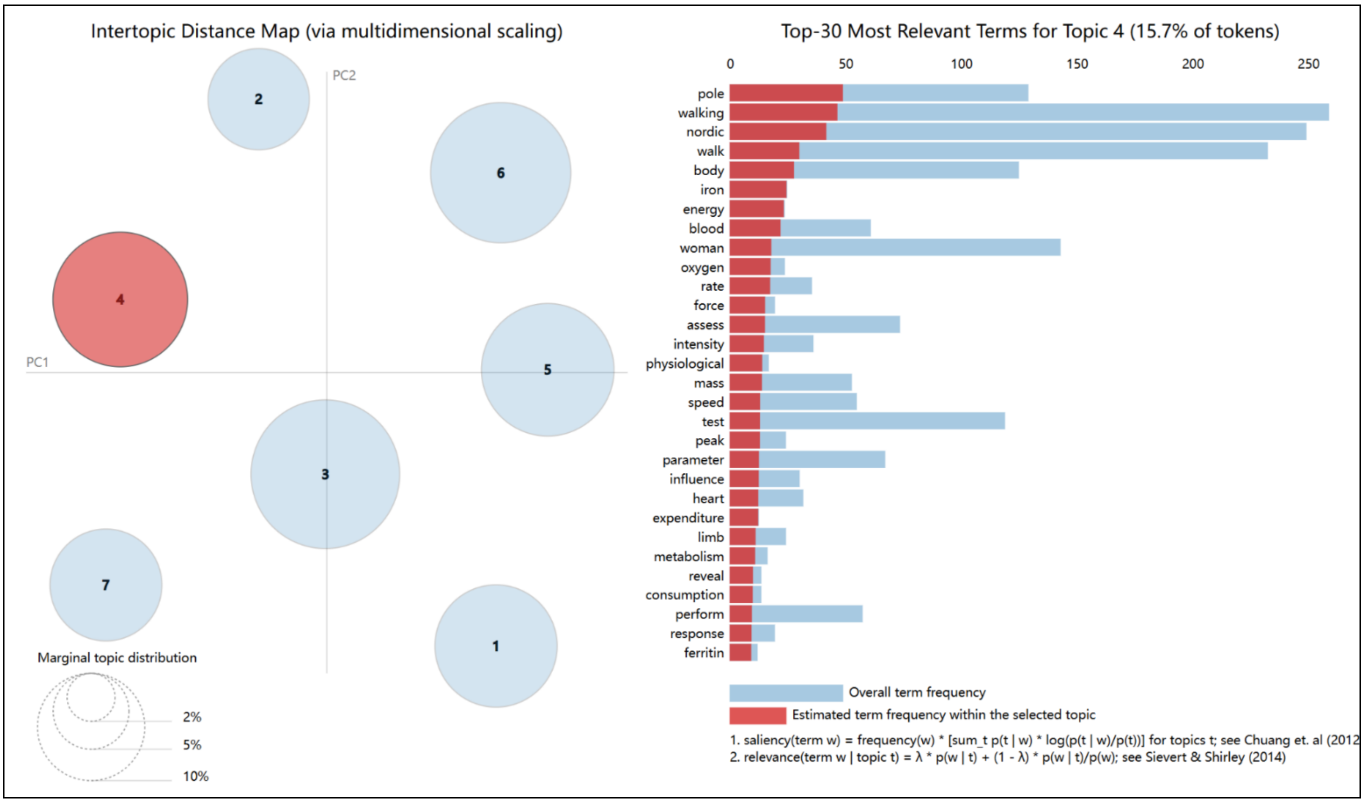

Supplementary Figure S1D. Topic 4: Exercise physiology and energy metabolism.

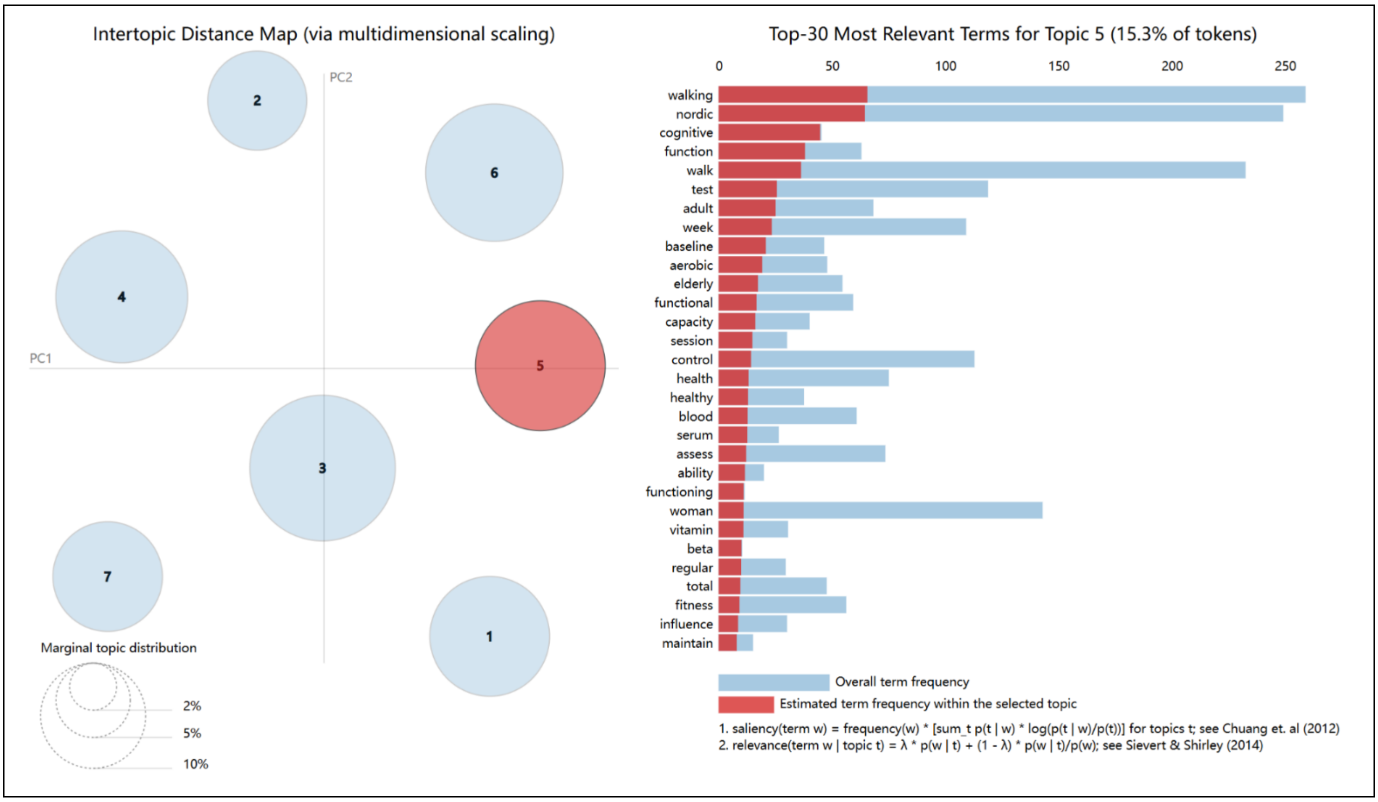

Supplementary Figure S1E. Topic 5: Cognitive function and health maintenance.

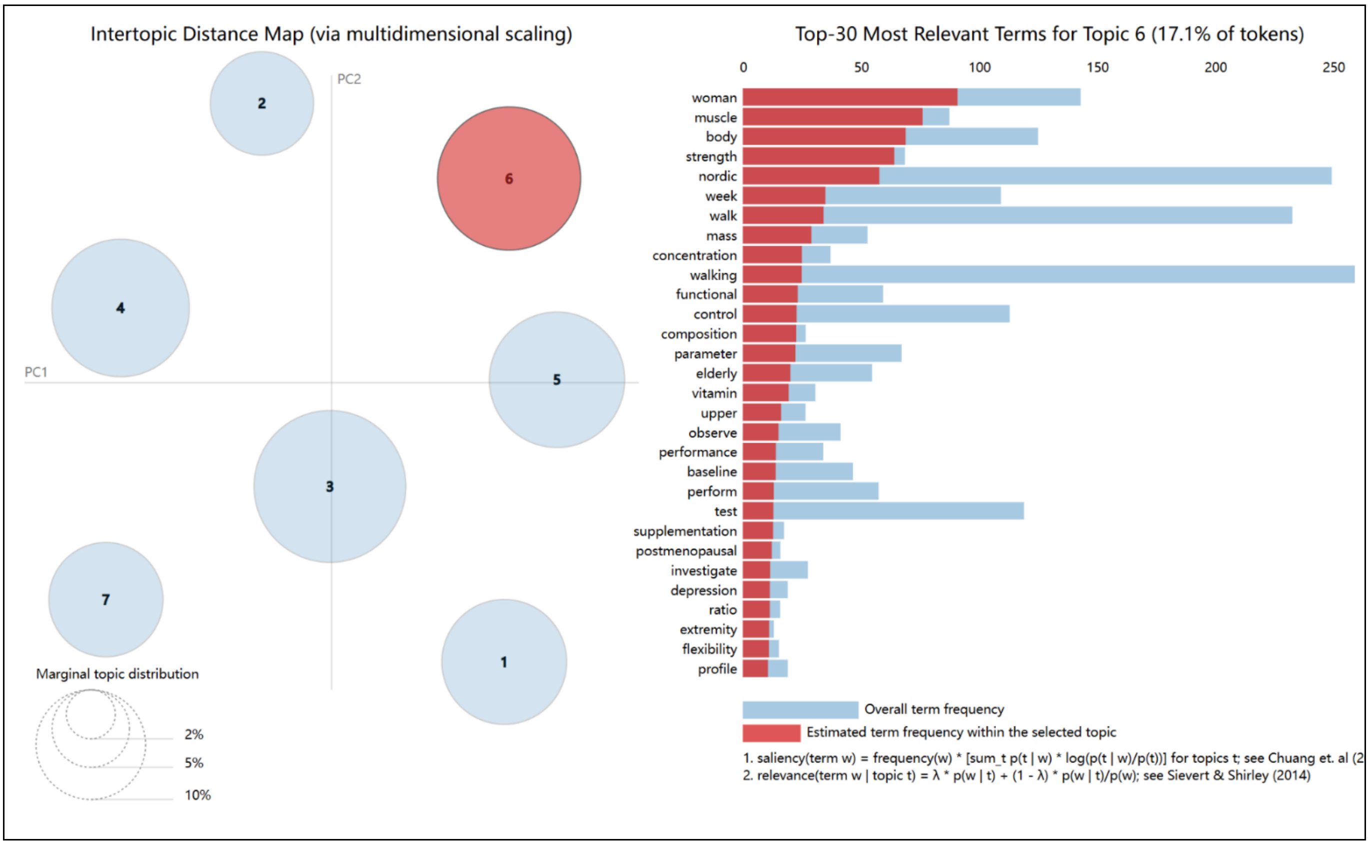

Supplementary Figure S1F. Topic 6: Body composition, muscle strength, and nutritional supplementation.

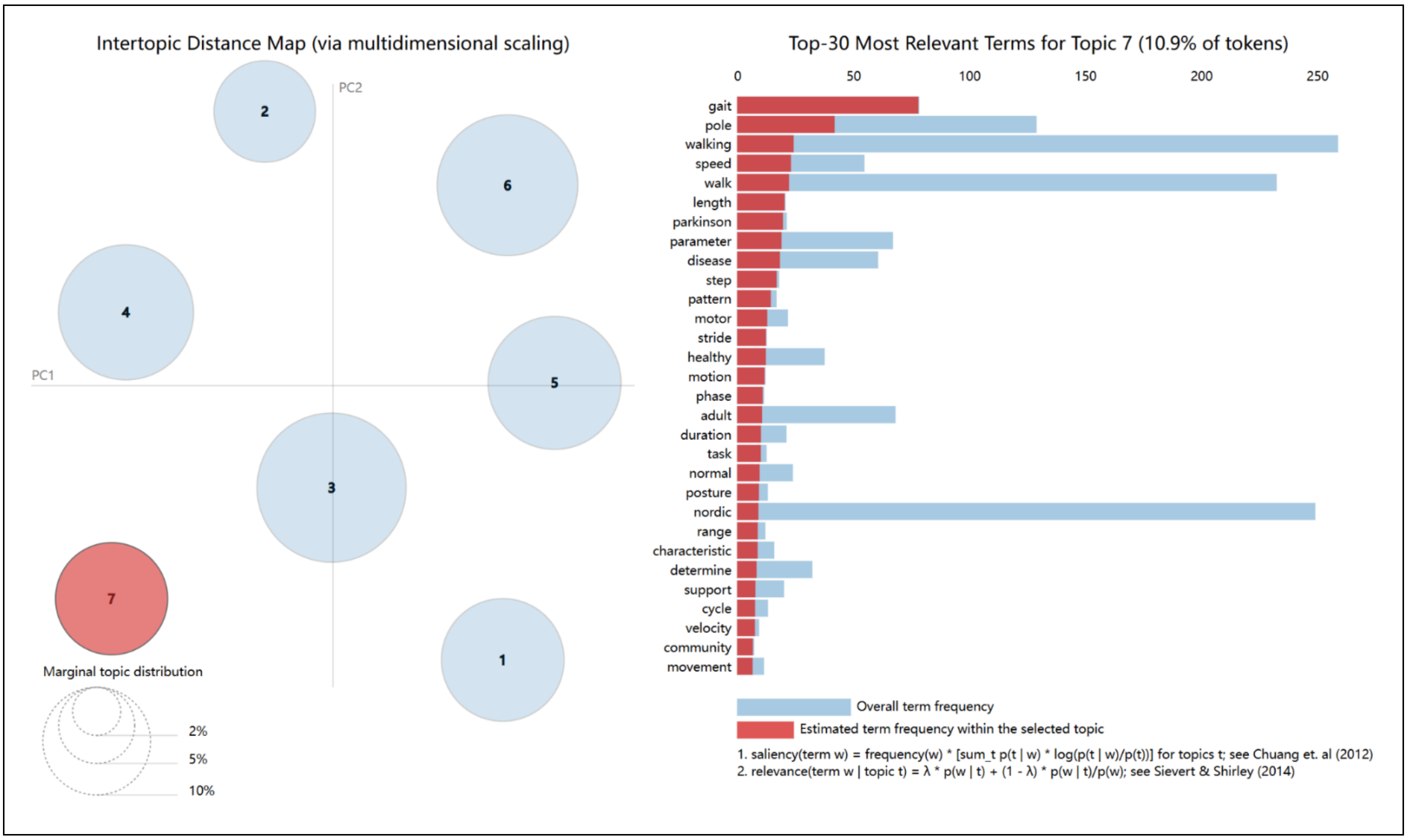

Supplementary Figure S1G. Topic 7: Gait parameters and Parkinson's disease rehabilitation.

Note. Each panel presents the intertopic distance map and the top relevant terms for the selected LDA topic. The red bubble indicates the selected topic, while blue bubbles indicate the remaining topics. Bubble size represents relative topic prevalence, and the distance between bubbles reflects semantic separation among topics. In the bar chart, red bars indicate the estimated term frequency within the selected topic, whereas blue bars indicate the overall term frequency in the corpus.
